# Cases from the Eye, Ear and Throat Clinic Atlanta Medical College

**Published:** 1887-10

**Authors:** 


### Clinic Reports.

### CASES FROM THE EYE, EAR AND THROAT CLINIC, ATLANTA MEDICAL COLLEGE.

(Reported for THE ATLANTA MEDICAL AND SURGICAL JOURNAL.)

A. C., æt. four years, admitted Thursday, December 6, 1883. A slight abrasion was observed on the left lid. There was much swelling of the lids and a profuse discharge of pus from the left eye. There was greatly the appearance of purulent ophthalmia. Everting the lid, however, a foreign body was discovered, which, being removed, was found to be a splinter about three-fourths of an inch long, and imbedded in the cellular tissue between the ball and the walls of the orbit. The splinter had been in the eye since the Tuesday before. Instructions were given for the eye to be bathed frequently with cold water and for three or four drops of a solution of atrop. sulph. (gr. j. ad aq. dest., ℥i) to be dropped in the eye three or four times a day. Under this treatment the patient recovered without any bad sequelæ in a few weeks.

C. P., æt. twenty years, admitted November 30, 1883. About two weeks previous, while cutting pine, she was struck in the left eye. A small body was found centrally located on the cornea. There are several ways to remove such a foreign body as this from the cornea. One of the difficulties to be overcome is to steady the ball. We may insert an eye speculum, and, if the patient will hold steady, pick out the body with a scoop. Or, again, without the speculum, we may keep the lids opened and steady the ball with the ring in a key or the handle of a pair of scissors. Again, we may hold the lids open with the thumb and index finger of one hand and pick out the body with the scoop. Or, as was done in this case, hold the lids open with the thumb and middle finger of one hand, and steady the ball with the index finger of the same hand, then with the scoop in the other hand gently pick out the body. The epithelium was necessarily destroyed in the operation, but was soon repaired. No after-treatment was necessary.

Mrs. M. E. C., æt. 30 years, admitted October 26, 1883. She presented a tumor on the right lower lid, which gave her little trouble, except when she took cold, when it became inflamed and painful. On examination it was found to be a chalazion, involving several meibomian glands. The novice would think the easiest way to reach this tumor would be through the skin. Besides the disfiguring that results from such procedure, this plan will be found very difficult. Knowing the lesions that exist, and at the same time remembering the anatomy of the parts, we at once see that as the glands lie just immediately beneath the conjunctiva, the most practical method is to make the incision through this membrane.

The lids in this case were everted, when a longitudinal slit was made over the tumor through the conjunctiva. The parts were then pressed as in opening a boil, when the contained cheese-like matter escaped. By a number of such pressures the remaining matter was removed. The patient was ordered to bathe both eyes frequently with cold water for a few days. In addition to this the patient was given a mercurial ointment (hydrg. ox. flav. gr. ss., ad unguent, petrol. ℥i). She was directed to take, on going to bed at night, a piece of this ointment, about the size of a small pea, and place it on the inner side of the lower lid of the affected eye, and rub it about, putting the finger on the ouside of the lid.

The patient required no further attention, and has been entirely freed from the tumor, the affected glands being, of course, destroyed.

